# Supplementary material for: Cognitive Effects of Simulated Galactic Cosmic Radiation Are Mediated by ApoE Status, Sex, and Environment in APP Knock-In Mice
Source: Int J Mol Sci. 2024 Aug 29;25(17):9379. doi: 10.3390/ijms25179379 (PMC11394682; doi:10.3390/ijms25179379)
Supplement: Supplementary file 1 [file ijms-25-09379-s001.zip › ijms-3185191-supplementary.pdf]

International Journal of *Molecular Sciences*

MDPI

Supplementary Materials

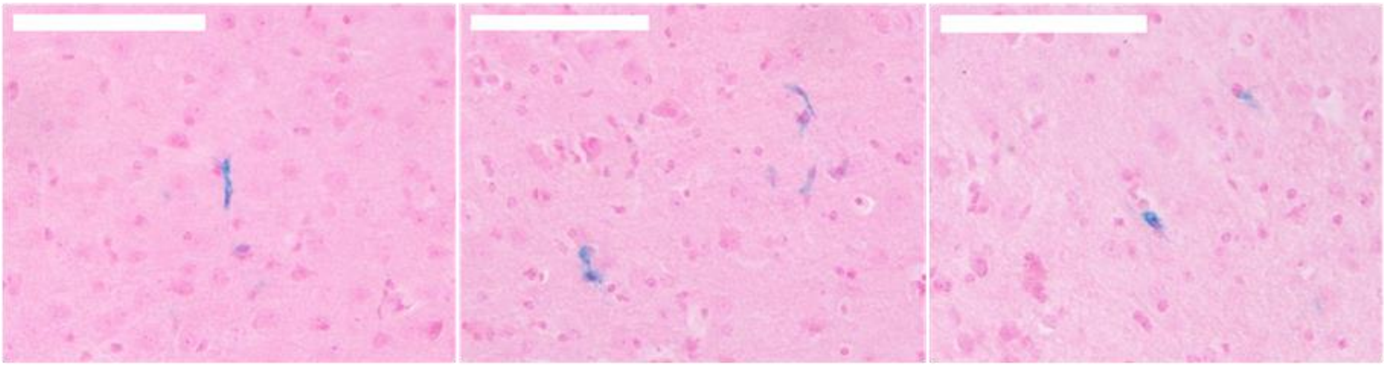

Supplementary Figure S1. Representative images of Prussian blue staining for hemosiderin deposits across the whole hemibrain section. Size of scale bars is 100 μm.

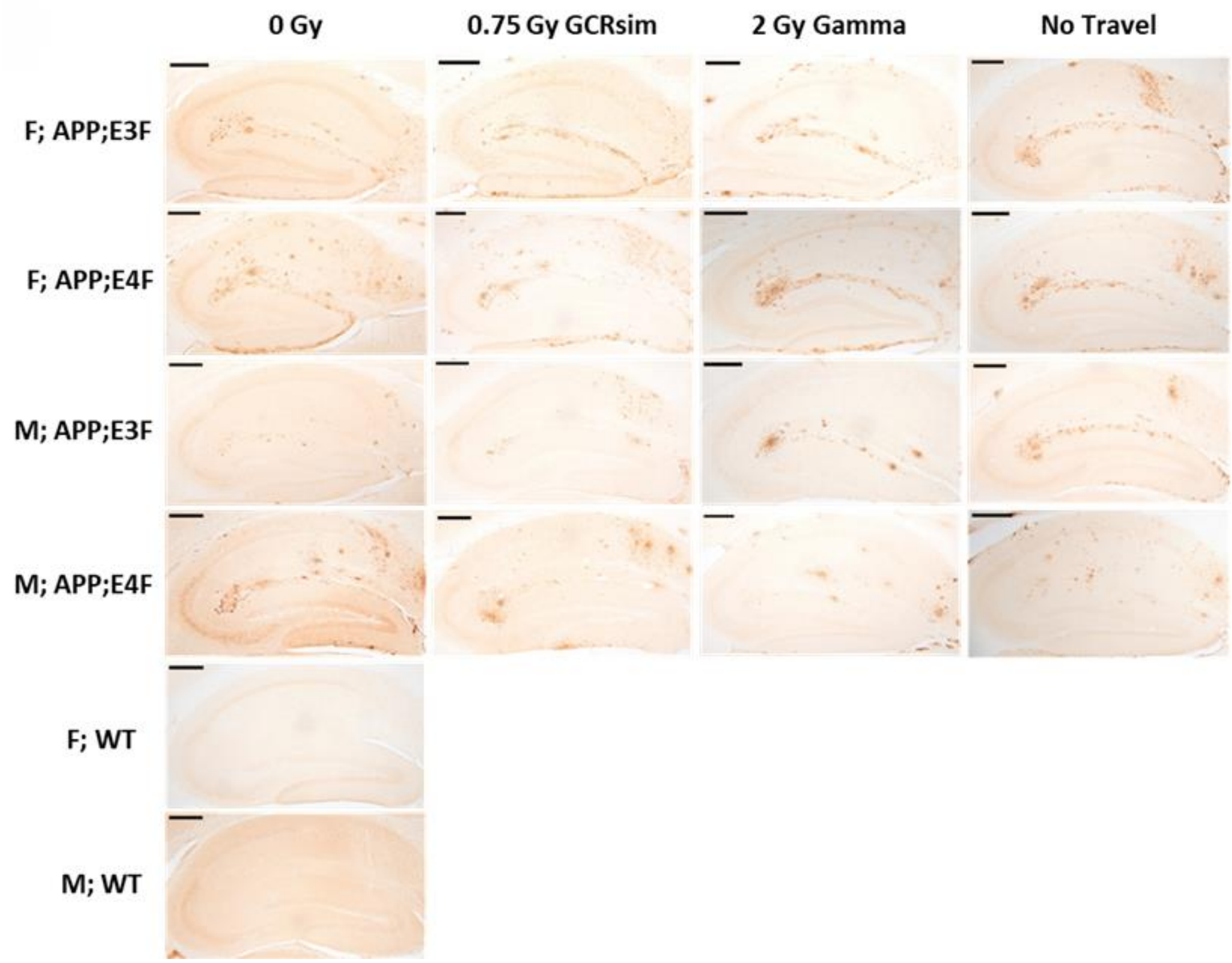

Supplementary Figure S2. Representative images for each group of S97 Aβ immunohistochemical staining for assessment of total amyloid beta load in the hippocampus. Size of scale bars is 300 μm.

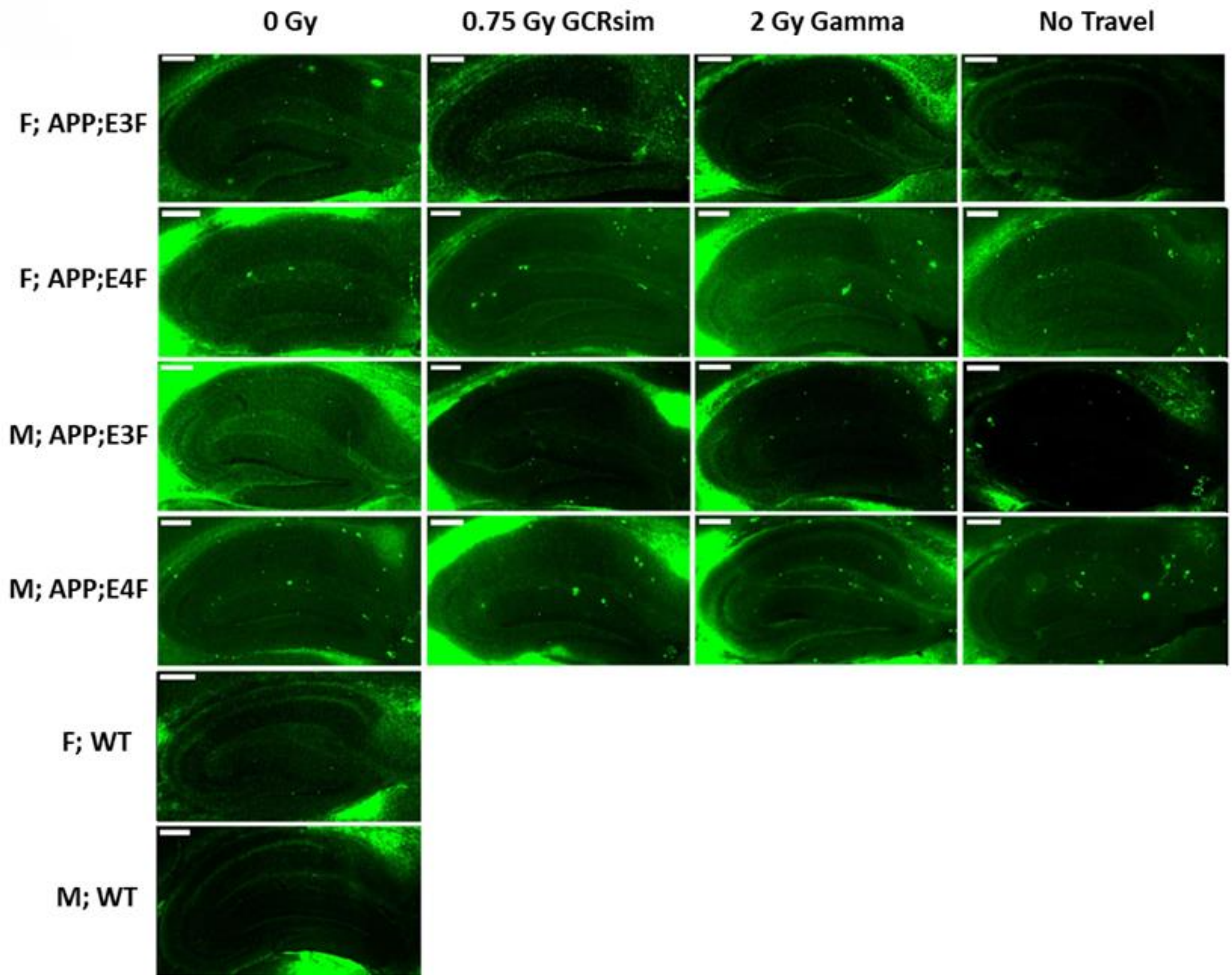

Supplementary Figure S3. Representative images for each group of Thioflavin S staining for assessment of fibrillar amyloid in the hippocampus. Size of scale bars is 300 μm.

Supplementary Table S1. Number of mice used for Open Field.

**Genotype**	**F, WT**	**F, APP;E3F**	**F, APP;E4F**	**M, WT**	**M, APP;E3F**	**M, APP;E4F**
No Travel	-	4	3	-	4	2
0 Gy (sham)	4	6	5	4	8	6
0.75 Gy GCRsim	-	8	7	-	8	7
2 Gy Gamma	-	5	8	-	7	7

Supplementary Table S2. Number of mice used for Rotarod.

**Genotype**	**F, WT**	**F, APP;E3F**	**F, APP;E4F**	**M, WT**	**M, APP;E3F**	**M, APP;E4F**
No Travel	-	4	3	-	4	2
0 Gy (sham)	4	6	5	4	8	6
0.75 Gy GCRsim	-	8	7	-	8	7
2 Gy Gamma	-	5	8	-	7	7

Supplementary Table S3. Number of mice used for Novel Object Recognition.

Genotype	F, WT	F, APP;E3F	F, APP;E4F	M, WT	M, APP;E3F	M, APP;E4F
No Travel	-	4	2	-	4	2
0 Gy (sham)	4	6	4	4	7	6
0.75 Gy GCRsim	-	8	7	-	8	7
2 Gy Gamma	-	5	7	-	7	6

Supplementary Table S4. Number of mice used for Spatial Novelty Y Maze.

Genotype	F, WT	F, APP;E3F	F, APP;E4F	M, WT	M, APP;E3F	M, APP;E4F
No Travel	-	4	2	-	4	2
0 Gy (sham)	4	6	4	4	8	5
0.75 Gy GCRsim	-	8	7	-	8	7
2 Gy Gamma	-	5	8	-	7	6

Supplementary Table S5. Number of mice used for Assessment of microhemorrhages.

Genotype	F, WT	F, APP;E3F	F, APP;E4F	M, WT	M, APP;E3F	M, APP;E4F
No Travel	-	4	3	-	3	2
0 Gy (sham)	3	6	4	4	8	6
0.75 Gy GCRsim	-	8	7	-	7	7
2 Gy Gamma	-	5	7	-	7	7

Supplementary Table S6. Number of mice used for Assessment of hippocampal S97.

Genotype	F, WT	F, APP;E3F	F, APP;E4F	M, WT	M, APP;E3F	M, APP;E4F
No Travel	-	4	3	-	4	2
0 Gy (sham)	4	6	5	4	8	6
0.75 Gy GCRsim	-	8	7	-	8	7
2 Gy Gamma	-	5	8	-	7	7

Supplementary Table S7. Number of mice used for Assessment of hippocampal Thioflavin S.

Genotype	F, WT	F, APP;E3F	F, APP;E4F	M, WT	M, APP;E3F	M, APP;E4F
No Travel	-	4	3	-	4	2
0 Gy (sham)	4	6	5	4	8	5
0.75 Gy GCRsim	-	8	7	-	8	7
2 Gy Gamma	-	5	8	-	7	7

Supplementary Table S8. Number of mice used for Assessment of HDL and total cholesterol, and triglycerides.

Genotype	F, WT	F, APP;E3F	F, APP;E4F	M, WT	M, APP;E3F	M, APP;E4F
No Travel	-	4	3	-	4	2
0 Gy (sham)	4	6	5	4	8	5
0.75 Gy GCRsim	-	8	7	-	8	7
2 Gy Gamma	-	5	8	-	7	7

Supplementary Table S9. Number of mice used for Assessment of cytokines.

Genotype	F, WT	F, APP;E3F	F, APP;E4F	M, WT	M, APP;E3F	M, APP;E4F
No Travel	-	4	3	-	4	2
0 Gy (sham)	4	6	5	4	8	5
0.75 Gy GCRsim	-	8	7	-	8	7
2 Gy Gamma	-	5	8	-	7	7

Supplementary Table S10. Number of mice used for Assessment of GHCL ApoE.

Genotype	F, WT	F, APP;E3F	F, APP;E4F	M, WT	M, APP;E3F	M, APP;E4F
No Travel	-	4	3	-	4	2
0 Gy (sham)	4	6	5	4	8	5
0.75 Gy GCRsim	-	8	7	-	8	7
2 Gy Gamma	-	5	8	-	7	7

Supplementary Table S11. Number of mice used for Assessment of GHCL Aβ.

Genotype	F, WT	F, APP;E3F	F, APP;E4F	M, WT	M, APP;E3F	M, APP;E4F
No Travel	-	4	3	-	4	2
0 Gy (sham)	4	6	5	4	8	5
0.75 Gy GCRsim	-	8	7	-	8	7
2 Gy Gamma	-	5	8	-	7	7
